# The effect of *Rhodiola rosea* supplementation on endurance performance and related biomarkers: a systematic review and meta-analysis

**DOI:** 10.3389/fnut.2025.1645346

**Published:** 2025-09-25

**Authors:** Xiaolin Wang, Xuezhen Yang, Zhendong Gao, Jin Zeng, Yutong Liu

**Affiliations:** ^1^Department of Physical Education, Ludong University, Yantai, China; ^2^Faculty of Educational Studies, Universiti Putra Malaysia, Serdang, Malaysia; ^3^Department of Physical Education, Soongsil University, Seoul, Republic of Korea; ^4^Department of Sports Teaching and Research, Lanzhou University, Lanzhou, China

**Keywords:** roseroot, physical endurance, oxidative stress, muscle damage, inflammation

## Abstract

This meta-analysis aimed to evaluate the effects of *Rhodiola rosea* L. (RR) supplementation on endurance performance and key physiological biomarkers, including oxidative stress, muscle damage, inflammation, and metabolic markers. A systematic search of Web of Science, PubMed, Scopus, EBSCO, and CNKI identified randomized controlled trials published up to March 20, 2025. Random-effects meta-analyses were conducted using R software, and methodological quality was appraised using the Physiotherapy Evidence Database scale. Additionally, subgroup analyses assessed the moderating effects of daily RR dosage, training duration, and training status. A total of 26 randomized controlled trials were included, involving 668 healthy participants with a mean age of 22.0 ± 10.7 years. The mean intervention duration was 33 days, with outcome assessments conducted from immediate post-exercise through 24-hour follow-up. The results indicated that RR supplementation significantly improved endurance-related outcomes, including VO_2max_ (11 studies; ES = 0.32, *p* < 0.01), time to exhaustion (TTE; 7 studies; ES = 0.38, *p* < 0.05), and time trial performance (TTP; 5 studies; ES = −0.40, *p* < 0.05). Antioxidant capacity was enhanced, with increases in total antioxidant capacity (TAC; 6 studies; ES = 0.59, *p* < 0.05) and superoxide dismutase (SOD; 7 studies; ES = 1.16, *p* < 0.01), alongside reductions in malondialdehyde (MDA; 6 studies; ES = −1.21, *p* < 0.001). RR also reduced creatine kinase (CK; 9 studies; ES = −0.84, *p* < 0.01) and lactate levels (LA; 7 studies; ES = −0.87, *p* < 0.05), indicating improved metabolic efficiency. No significant effects were observed on inflammatory markers, including interleukin-6 (IL-6) and C-reactive protein (CRP). Subgroup analyses indicated greater VO_2*max*_ improvements at doses >600 mg/day, with trained individuals exhibiting lower CK levels and more pronounced reductions in CK at early follow-up assessments (≤15 min) post-exercise. In conclusion, RR supplementation is an effective ergogenic aid for enhancing endurance performance and improving physiological biomarkers related to oxidative stress, muscle damage, and metabolic efficiency, though heterogeneity across studies warrants cautious interpretation.

**Systematic review registration:**

https://www.crd.york.ac.uk/prospero/, PROSPERO CRD42024619014.

## 1 Introduction

*Rhodiola rosea* L. (RR) is a perennial herb native to cold, high-altitude regions of the Northern Hemisphere, traditionally used in Tibetan, Russian, and Chinese medicine to enhance physical and mental well-being (1–3). As a recognized adaptogen, RR is widely included in dietary supplements for its ability to improve physiological responses to physical and psychological stress. Its key bioactive compounds, rosavin and salidroside, have been reported to reduce fatigue, enhance energy metabolism and oxygen utilization, and mitigate oxidative stress (4–6). These properties have garnered increasing attention regarding RR's potential to enhance endurance capacity, recovery, and long-term training adaptations (7–9).

Multiple mechanisms have been proposed to elucidate the ergogenic effects of RR supplementation on endurance performance, recovery, and long-term training adaptations. Evidence from rodent studies underscores RR's capacity to significantly enhance endurance performance (10–12). This enhancement is attributed to the multifaceted modulation of various physiological processes, including the reduction of muscle damage, alleviation of oxidative stress, suppression of inflammatory responses, and optimization of energy metabolism (13–15). Specifically, RR has been shown to reduce key markers of muscle damage (e.g., lactate dehydrogenase and creatine kinase [CK]), while modulating oxidative stress (e.g., lowering malondialdehyde [MDA] levels and enhancing the activity of antioxidant enzymes such as superoxide dismutase [SOD] and catalase) (5, 16). Moreover, its antioxidative properties counteract free radical-mediated cellular injury, promoting efficient post-exercise repair (5). RR supplementation also demonstrates potent anti-inflammatory effects, significantly reducing inflammatory biomarkers such as C-reactive protein (CRP), interleukin-6 (IL-6), interleukin-8 (IL-8), interleukin-1 beta (IL-1β), and tumor necrosis factor-alpha (TNF-α), thereby alleviating exercise-induced inflammation and expediting recovery (13, 15). Additionally, by enhancing energy metabolism and lactate clearance, RR supplementation improves mitochondrial function and oxygen utilization, effectively delaying fatigue and bolstering endurance capacity (4, 6). These beneficial effects are likely mediated by its ability to stimulate mitochondrial biogenesis, improve ATP synthesis efficiency, and optimize muscle metabolic pathways (10, 17). In summary, these findings underscore the robust capacity of RR supplementation to enhance endurance performance and recovery through integrative mechanisms targeting muscle damage, oxidative stress, inflammation, and energy efficiency, supported by robust evidence from rodent studies.

Despite the significant benefits demonstrated in animal studies, human research results remain inconsistent. Some studies suggest that RR supplementation can enhance endurance performance, such as extending time to exhaustion (TTE), improving VO_2max_, and affecting related biomarkers (e.g., reducing muscle damage, alleviating oxidative stress, and decreasing inflammation) (9, 18, 19). However, other studies have failed to observe these effects (20–22). Shanely et al. (22) reported no significant differences between the RR supplementation and control groups in marathon performance, and interleukin levels among marathon runners. Parisi et al. (21) found that RR supplementation did not significantly affect time to TTE or VO_2max_ in trained male athletes compared to the control group, although it did lower lactate and CK levels. Similarly, Jówko et al. (20) observed no improvements in endurance capacity or hormonal levels in healthy male students, but reported a significant increase in total antioxidant capacity (TAC).

Although two systematic reviews have assessed the impacts of RR supplementation on exercise performance and related biomarkers (6, 7), the studies included in these reviews were limited, and neither employed meta-analysis techniques. This lack of statistical rigor restricts the ability to draw robust conclusions. These reviews primarily focused on overall exercise performance, while the majority of the primary studies have concentrated on endurance performance. Given the discrepancies in existing literature, this study aims to conduct a comprehensive meta-analysis to systematically evaluate the effects of RR supplementation on human endurance performance and related biomarkers. It is hypothesized that RR supplementation will significantly enhance endurance performance compared to a placebo or control group.

## 2 Materials and methods

This systematic review was conducted in accordance with the PRISMA guidelines for systematic reviews (52) and meta-analyses and is registered with Prospero (registration number: CRD42024619014).

### 2.1 Data sources and search strategy

A comprehensive literature search was conducted by two independent researchers across multiple databases, including Web of Science, PubMed, Scopus, EBSCO MEDLINE databases, and CNKI, for articles published up to March 20, 2025. The search strategy involved using the following combinations of terms: *Rhodiola rosea*, Rhodiola, Rosea, Roseroot, Golden root, Arctic root, Rhodioloside, Salidroside, Endurance, Sport, Athletic, Exercise, and Training. Detailed search alerts are documented in Supplementary File 1. The identified articles were managed and screened using EndNote reference management software. Duplicates were first removed using EndNote. The titles and abstracts of the remaining articles were then independently reviewed by the two researchers. Discrepancies between the two researchers were resolved by a third researcher.

### 2.2 Selection criteria

Studies eligible for inclusion were required to meet the following PICOS criteria: healthy participants aged 18–50 years without underlying diseases (P), with RR supplementation of no fixed duration as the intervention (I), compared to a placebo or control group (C). The outcomes (O) included endurance performance (e.g., TTE, TTP, VO_2*max*_) and related physiological biomarkers (e.g., muscle damage biomarkers, oxidative stress markers, inflammation markers, lactate levels). Studies were required to be randomized controlled trials (RCTs), including double-blind, cross-over, and parallel-group designs (S). For further details on the PICOS criteria, please refer to Table 1. Exclusion criteria included: (1) studies that did not report specific outcome data; (2) editorial articles, conference abstracts, and reviews; (3) studies with a PEDro scale rating of <5.

**Table 1:** PICOS (Population Intervention Control Outcome Study) criteria.

Population	Healthy participants aged 18–50 years without underlying diseases
Intervention	RR supplementation with no fixed duration
Comparison	Placebo or no supplementation (control group)
Outcome	Endurance performance (such as time to exhaustion, time trial performance, or VO_2max_) or related physiological biomarkers (e.g., Muscle damage biomarkers, oxidative stress biomarkers, inflammation biomarkers, or lactic acid levels)
Study design	Randomized controlled trials (RCTs), including double-blind, cross-over, and parallel-group designs

### 2.3 Data extraction

The following information was extracted from the included studies: study source (authors, publication year), study design (parallel or cross-over), participant characteristics (gender, age, sample size of each group), supplementation protocol (type, dosage, duration), exercise intervention or testing methods, and outcome variables (mean and standard deviations). The primary outcome variables included: aerobic performance (VO_2max_, TTE, time trial performance [TTP]), muscle damage markers (CK), oxidative stress markers (TAC, MDA, and SOD), inflammatory markers (IL-6, and CRP), and metabolic indicators (lactate levels [LA]).

### 2.4 Quality assessment and risk of bias

The methodological quality of the included studies was assessed using the Physiotherapy Evidence Database scale (PEDro; https://www.pedro.org.au). Following the approach used in previous meta-analyses (23), studies were classified as follows: low quality (≤ 3 points), moderate quality (4–5 points), and high quality (6–10 points). The risk of bias across studies was evaluated using funnel plots and Egger's regression test to assess potential asymmetry and publication bias.

### 2.5 Data analysis

Meta-analysis was performed using the R packages (R version 4.3.0 with R Studio version 2023.06.1 + 524). The *metagen()* function from the meta package was used for meta-analyses and subgroup analyses. The standardized mean difference (SMD: Hedges' g) was used to assess the difference between RR supplementation and placebo/control groups. A random-effects model was applied, weighting studies by their standard error to address heterogeneity. Effect sizes were categorized as trivial (< 0.2), small (0.2–0.5), medium (0.5–0.8), and large (>0.8) (24). Heterogeneity was assessed with *I*^2^ and τ^2^, where *I*^2^ values of ≤ 25%, 25%−50%, and ≥75% indicate low, medium, and high heterogeneity, respectively. A total of 10 meta-analyses were conducted, assessing the following outcomes: (1) aerobic performance (TTP, TTE, VO_2max_); (2) muscle damage biomarkers (CK); (3) oxidative stress biomarkers (TAC, MDA, SOD); (4) inflammation biomarkers (IL-6, CRP); and (5) metabolic markers (LA).

Additionally, subgroup analyses were conducted to evaluate the moderating effects of training-related factors, including daily RR dosage, training duration, follow-up time points, training status, and comparator type, on the observed outcomes. These analyses were restricted to cases where at least two subgroups included three or more relatively homogeneous studies. Statistical significance was determined at a threshold of *p* < 0.05.

## 3 Results

### 3.1 Search results and general characteristics of participants and protocols

**Figure 1:**
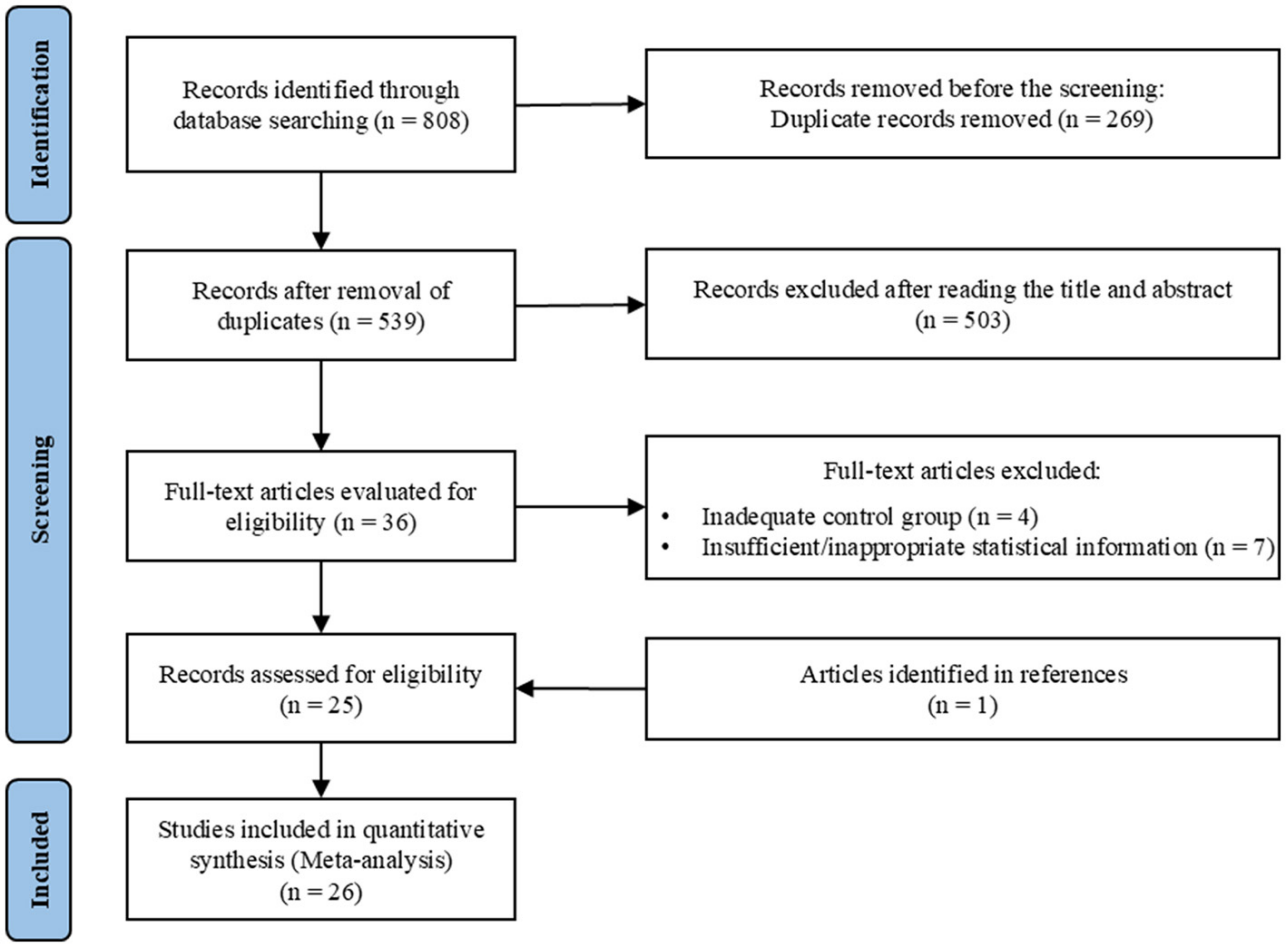
Flow diagram of literature search.

A total of 808 articles were initially identified from various databases: Scopus (*n* = 199), EBSCO Medical Databases (*n* = 161), Web of Science (*n* = 122), PubMed (*n* = 87), and CNKI (*n* = 239). After removing duplicates, 539 articles remained for title and abstract screening. Following this, 36 full-text articles were assessed for eligibility. Ultimately, 26 studies were included in the meta-analysis (see Figure 1).

The studies included in the meta-analysis are summarized in Table 2. A total of 668 participants from healthy populations were involved, with ages ranging from 11 to 45 years. Among the 26 studies, 15 focused on male participants, 3 on female participants, and 8 included both male and female. The training durations ranged from 3 days to around 3 months. Follow-up assessments ranged from within 15 min post-exercise to fasting measurements taken the following morning, encompassing both acute and extended recovery phases. The studies included both trained (13 studies) and untrained participants (13 studies). Most studies involved aerobic and endurance training, although some did not provide detailed descriptions of the training protocols. The tests assessed endurance performance and related physiological biomarkers, including TTP (*n* = 5 groups), TTE (*n* = 7 groups), VO_2max_ (*n* = 11 groups), CK (*n* = 9 groups), TAC (*n* = 6 groups), MDA (*n* = 6 groups), SOD (*n* = 7 groups), IL-6 (*n* = 3 groups), CRP (*n* = 3 groups), and LA (*n* = 7 groups).

**Table 2:** Characteristics of the included studies.

**Study**	**Design**	**Subjects; *N* (SUP, PLA); age**	**Supplementation (mg/d)**	**Training, testing and blood protocol**	**Comparator type**	**Outcomes**
Abidov et al. (35)	Double-blind RCT	Untrained volunteers; 12, 12; 21–24 years	680 mg RR (30 d); Controlled dietary intake	Not reported; Incremental cycle ergometer tests; 5 h post-exercise	Placebo	CK: SUP vs. PLA ↓2.4% CRP: SUP vs. PLA ↓46.2%;
Chen et al. (18)	Double-blind RCT	Male long-distance athletes; 9, 9; 19.7 ± 0.2 years	2000 mg RR and Cordyceps sinensis (14 d); Controlled dietary intake	Running, intervals, strength, and basketball, 2 times/week; Bruce incremental treadmill test; Fasting	Placebo; Starch capsule	TTE: SUP↑0.7%, PLA↑0.3%; VO_2max_: SUP↓0.8%, PLA↓4.3%
Cui et al. (36)	RCT	Male soldiers; 10, 10; 19–21 years	500 mg RR (6 d)	Not reported; 5-min step test; 5min post-exercise	Placebo; Starch capsule	SOD: SUP↑19.7%, PLA↓0.8%; CK: SUP↓20.5%, PLA↓1.1%; MDA: SUP↓26%, PLA↓1%
De Bock et al. (19)	Double-blind crossover RCT	Physically active adults; 12, 12; 21 ± 0.3 years	200 mg RR during fasting (28 d)	Not reported; Incremental cycle ergometer tests; 2 min post-exercise	Placebo; Starch capsule	TTE: SUP vs. PLA ↑2.4%; VO_2max_: SUP↓1.1%, PLA↓1.4%; LA: SUP↓7%, PLA↑7.7%
Duncan et al. (37)	Double-blind crossover RCT	Male recreational exercisers; 12, 12; 24.6 ± 6 years	Single dose, 203 mg (3 mg/kg) 60-min pre-exercise	Recreational physical activity, 3–10 times/week; Jones incremental treadmill test; Immediately post-exercise	Placebo; Maltodextrin	5km run time: SUP vs. PLA ↓3.3%
Gao and Zhang (38)	Double-blind RCT	Track and field, wrestling, and judo athletes; 30, 33; 18.6 ± 0.7 years	250 ml RR (40 d)	Not reported; Cycle-ergometer test	Placebo; Matched beverage	VO_2max_: SUP↑7.2%, PLA↑0.6%
He (53)	Double-blind RCT	Male endurance runner; 10, 10; 19.2 ± 0.6 years	7.6 mg Rhodioloside (15 d)	Not reported; Incremental cycle ergometer test; Immediately post-exercise	Placebo; Matched beverage	TTE: SUP vs. PLA↑3.4%; MDA: SUP vs. PLA↓11.9%; SOD: SUP vs. PLA↑5.4%
Jia et al. (39)	Crossover RCT	Male marathon athletes; 8, 8; 42.5 ± 2.2 years	600 mg RR (30 d)	200–300 km running per month; 20 km running test; Immediately post-exercise	Control	CK: SUP vs. PLA ↓27.9%; SOD: SUP vs. PLA ↑6.7%; TAC: SUP vs. PLA ↑13.3%; MDA: SUP vs. PLA ↓22.7%
Jówko et al. (20)	Double-blind RCT	Male physical education students; 13, 13; 20.7 ± 0.3 years	600 mg RR extract (28 d); Controlled dietary intake	Not reported; Incremental cycle ergometer test; 3 min post-exercise	Placebo; Placebo tablet	TTE: SUP↑2.6%, PLA↓1.3%; VO_2max_: SUP↓2.8%, PLA↓1.8%; TAC; SUP↑5.5%, PLA↑5.2%; SOD: SUP↓1.9%, PLA↓18.2%; LA: SUP↓6.7%, PLA↑8.6%
Kreipke et al. (40)	Double-blind RCT	Active college-aged men; 10, 11; 22 ± 2.3 years	930 mg RR and Cordyceps sinensis (98 d); Controlled dietary intake	Resistance training and HIIT training, 2 times/week; Incremental treadmill test; Fasting	Placebo; Dextrose	VO_2max_: SUP↑2.4%, PLA↓2.4%
Liao et al. (41)	Double-blind RCT	Young sedentary individuals; 7, 7; 21.4 ± 0.4 years	1,060–1,800 mg RR (56 d); Controlled dietary intake	Cycle ergometer training at 60–75% maximal work rate, 3 times per week; Cycle ergometer test; Fasting	Placebo; Sweetener,	TAC: SUP↑0%, PLA↓2%; VO_2max_: SUP↑11.6, PLA↑8.9%
Lin et al. (42)	Double-blind crossover RCT	Active male university students; 12, 12; 24.7 ± 0.5 years	800 mg (8 d)	30 min run at 75% VO_2max_, 3 times/week; Bruce incremental treadmill test; 1h post-exercise	Placebo; HPMC capsule	CK: SUP↑64.7%, PLA↑68.7%; IL-6: SUP↑25.5%, PLA↑26.7
Noreen et al. (43)	Double-blind crossover RCT	Recreationally active college women; 15, 15; 21.3 ± 0.1 years	3 mg/kg RR, Single dose	Not reported; 6-mile cycle ergometer test; 2 min post-exercise	Placebo; Maltodextrin	TOC: SUP vs. PLA ↓1.6%
Noreen et al. (44)	Double-blind crossover RCT	Recreationally active college women; 18, 18; 22 ± 3.3 years	3 mg/kg RR, Single dose	Not reported; 6-mile cycle ergometer test; 2 min post-exercise	Placebo; Maltodextrin	TOC: SUP vs. PLA ↓1.6%;
Parisi et al. (45)	Double-blind crossover RCT	Well-trained male athletes; 34, 34; 24.5 ± 3.2 years	170 mg RR (28 d); Controlled dietary intake	Endurance training; Cycle-ergometer test	Placebo	TTE: SUP vs. PLA ↑5.5%; VO_2max_: SUP vs. PLA ↑3.1%
Parisi et al. (21)	Double-blind crossover RCT	Well-trained male athletes; 14, 14; 25 ± 5 years	170 mg RR (28 d); Controlled dietary intake	Endurance training; Cycle-ergometer test; Immediately post-exercise	Placebo	TTE: SUP vs. PLA ↑0%; VO_2max_: SUP vs. PLA ↑5.5%; CK: SUP vs. PLA ↓36.8%; TAC: SUP vs. PLA ↓1.7%; MDA: SUP vs. PLA ↑11%; LA: SUP vs. PLA ↓4.9%; IL-6: SUP↑2.6%, PLA↑68.6%
Qiao (46)	RCT	Male track and field athletes; 20, 20; 17.7 ± 2.7 years	1,500 mg/kg RR herbal medicine (60 d)	Normal track and field training; Not reported; Fasting	Placebo	SOD: SUP↑17.6%, PLA↓23.7%; TAC: SUP↑36.2%, PLA↓3.4%; MDA: SUP↓20.5%, PLA↑23.6%
Schwarz et al. (8)	Double-blind RCT	Healthy active young adults; 25, 24; 21.1 ± 3.9 years	60 mg salidroside (16 d); Controlled dietary intake	Running or jogging at least 1 time/week; Graded exercise test on a treadmill; Fasting	Placebo; Rice flour	VO_2max_: SUP↓1.5%, PLA↓3.3%; CRP: SUP↓9.8%, PLA↑0.3%; CK: SUP↑0.8%, PLA↓4.4%
Shanely et al. (22)	Double-blind RCT	Trained runners; 24, 23; 42.1 ± 1.3 years	600 mg RR extract (30 d)	Not reported; Marathon race test; 10 min post-exercise	Placebo; Starch capsule	CRP: SUP↓14.5%, PLA↑1.1%; IL-6: SUP vs. PLA ↓17.6%
Skarpanska-Stejnborn et al. (47)	Double-blind RCT	Male rowers; 11, 11; 21.2 ± 1.1 years	200 mg RR extract (28 d); Controlled dietary intake	Rowing training; 2 km rowing test; 1 min post-exercise	Placebo; Placebo capsule	Total running time: SUP↓1.6%, PLA↓1.4%; SOD: SUP↑12.6%, PLA↑3%; TAC: ↑19.3%, PLA↑3.2%; CK: SUP↓20%, PLA↑12%; LA: SUP↓1.1%, PLA↓8.5%
Song et al. (48)	RCT	Male student-athletes; 20, 20; 20.3 ± 3.3 years	250 g RR decocted into 500 ml, with 100 ml taken daily (5 d)	Not reported; 1 h cycle-ergometer at 75% VO_2max_; Fasting	Placebo; Matched beverage	SOD: SUP↓12.3%, PLA↓25.7%; MDA: SUP↓57.5%, PLA↓24.6%
Timpmann et al. (49)	Double-blind RCT	Male military students; 10, 10; 22.5 ± 3.1 years	432 mg RR (8 d)	Not reported; Incremental Treadmill Test; 50 min post-exercise	Placebo; Starch capsule	TTE: SUP↑78.3%, PLA↑72.3%; LA: SUP↓15.6%, PLA↑2.8%
Yun et al. (9)	RCT	Non-aerobically trained subjects; 12, 12; 20 ± 1.4 years	2,400 mg RR (30 d); Controlled dietary intake	3 × 35-min treadmill at 60–70% HR_max_ per week; Bruce incremental treadmill test	Placebo; Placebo capsule	5 km running time: SUP vs. PLA ↓15.2%; VO_2max_: SUP vs. PLA ↑6%
Zhang et al. (50)	Double-blind RCT	University students; 33, 34; 20 ± 1.2 years	1,080 mg RR, 2 capsules (49 d)	Not reported; Incremental cycle-ergometer test; Fasting	Placebo; Placebo capsule	VO_2max_: SUP↑6.6%, PLA↑0.6%;
Zhao (54)	Double-blind crossover RCT	Male amateur marathon runner; 8; 8; 42.7 ± 1.7 years	600 mg RR (30 d)	200 km marathon training per week; 20 km running test; 1 h post-exercise	Control	CK: SUP vs. PLA↓29.2%
Zheng and Liu (51)	RCT	Male physical education students; 10, 10; 21.9 ± 1.2 years	600 mg RR (28 d)	Aerobic gymnastics exercise at 22–28 beats/10 s, 4 times/week; 12 min post-exercise	Control	CK: SUP↑42.4%, PLA↑73.4%; LA: SUP vs. PLA↓14%

CK, creatinine kinase; CRP, C-reactive protein; IL-6, interleukin-6; LA, lactic acid; MDA, malondialdehyde; PLA, Placebo group; RR, Rhodiola rosea; SOD, superoxide dismutase; SUP, supplement group; TAC, total antioxidant capacity; TOC, time to completion; TTE, time to exhaustion.

### 3.2 Quality assessment of studies and risk of bias

The quality assessment of the included studies, based on the PEDro scale ratings, is presented in Supplementary File 2. Of the studies included in the meta-analysis, 2 were rated as moderate quality (scoring 4–5 points), while 24 studies were rated as good quality (scoring 6–9 points). The median PEDro score across the studies was 7 out of a possible 10 points. Overall, the high quality of the included studies supports the reliability and robustness of the meta-analysis results. Funnel plots for all outcome measures showed a generally symmetrical distribution, suggesting no significant publication bias (see Supplementary File 3).

### 3.3 Meta-analysis results

The overall effects of RR supplementation on endurance performance and related biomarkers are shown in Table 3, with forest plots displayed in Supplementary File 4. The findings indicated that RR supplementation produced significant improvements in endurance performance compared to placebo or control groups. Specifically, VO_2max_ was significantly increased (ES = 0.32, 95% CI [0.12, 0.52], *p* < 0.01), TTE was prolonged (ES = 0.38, 95% CI [0.07, 0.69], *p* < 0.05), and TTP improved (ES = −0.40, 95% CI [−0.78, −0.01], *p* < 0.05).

**Table 3:** Synthesized results of RR on exercise endurance performance and related biomarkers.

**Outcome variables**		***k***	**ES (95% CI)**	***p***	***I*^2^ (%)**	**RW (%)**
Endurance performance metrics	TTP	5	−0.40 (−0.78, −0.01)	0.043	24.8	16.3–25.9
	TTE	7	0.38 (0.07, 0.69)	0.016	14.5	9.6–29.9
	VO_2max_	11	0.32 (0.12, 0.52)	0.002	0	4.5–16.7
Muscle damage biomarkers	CK	9	−0.84 (−1.35, −0.34)	0.001	83.0	9.1–16.1
Oxidative stress biomarkers	TAC	6	0.59 (0.06, 1.13)	0.029	59.7	13.9–19.4
	SOD	7	1.16 (0.37, 1.94)	0.004	81.8	13.2–15.8
	MDA	6	−1.21 (−1.87, −0.55)	0.000	74.7	14.3–18.2
Inflammation biomarkers	IL-6	3	−1.50 (−3.51, 0.51)	0.143	91.9	31.8–34.5
	CRP	3	−1.34 (−3.22, 0.55)	0.166	90.7	30.9–34.6
Metabolic markers	LA	7	−0.87 (−1.55, −0.19)	0.012	77.5	13.7–15.8

CK, creatinine kinase; CRP, C-reactive protein; IL-6, interleukin-6; LA, lactic acid; MDA, malondialdehyde; RR, Rhodiola rosea; SOD, superoxide dismutase; SUP, supplement group; TAC, total antioxidant capacity; TTE, time to exhaustion; TTP, time trial performance.

Regarding muscle damage, RR supplementation led to a significant reduction in CK levels (ES = −0.84, 95% CI [−1.35, −0.34], *p* < 0.01). Furthermore, RR supplementation significantly modulated oxidative stress markers, enhancing TAC (ES = 0.59, 95% CI [0.06, 1.13], *p* < 0.05) and SOD levels (ES = 1.16, 95% CI [0.37, 1.94], *p* < 0.01), while simultaneously reducing MDA levels (ES = −1.21, 95% CI [−1.87, −0.55], *p* < 0.001), suggesting improved antioxidant activity and reduced oxidative damage.

Additionally, RR supplementation resulted in a significant reduction in post-exercise LA levels (ES = −0.87, 95% CI [−1.55, −0.19], *p* < 0.05). However, no significant effects were observed for the inflammatory biomarkers IL-6 (ES = −1.50, 95% CI [−3.51, 0.51], *p* = 0.143) and CRP (ES = −1.34, 95% CI [−3.22, 0.55], *p* = 0.166), compared to control groups.

### 3.4 Subgroup analysis results

The moderating effects of training factors on the impact of RR supplementation on endurance performance and related biomarkers are summarized in Table 4. The analysis revealed that RR's effect on VO_2max_ was significantly influenced by daily dosage, with higher doses (exceeding 600 mg/d) resulting in more substantial improvements compared to lower doses. Follow-up time points also significantly moderated the effect of RR supplementation on CK levels. Early follow-up assessments (≤ 15 min) were associated with markedly greater reductions in CK levels, whereas this effect diminished at later follow-up intervals. Additionally, training status was found to significantly moderate the effect of RR supplementation on muscle damage markers, particularly CK, with trained individuals exhibiting notably lower CK levels than their untrained counterparts. No other factors were found to significantly moderate the effects of RR supplementation on endurance performance or related physiological indicators.

**Table 4:** Moderation analysis of individual and training factors on the effects of LL-BFR vs. HLR on maximal strength, muscle power, and jump performance.

**Covariate**	***k***	***ES***	**95% CI**	***I*^2^ (%)**	***p*-value**
**Time to exhaustion (TTE)**					
**RR dosage**					
≤ 432 mg/d	4	0.19	−0.15, 0.53	0	0.102
>432 mg/d	3	0.77	0.16, 1.37	25.3	
**Training duration**					
≤ 28 d	3	0.36	0.17, 0.90	0	0.833
>28 d	4	0.44	−0.08, 0.96	53.0	
**Training status**					
Trained	4	0.20	−0.15, 0.54	0	0.142
Recreationally active	3	0.71	0.12, 1.30	32.1	
**Maximal oxygen uptake (VO**_2max_ **)**					
**RR dosage**					
≤ 600 mg/d	6	0.16	−0.08, 0.41	0	0.029
>600 mg/d	5	0.64	0.29, 0.98	0	
**Training duration**					
≤ 28 d	7	0.24	0.01, 0.48	0	0.246
>28 d	4	0.58	0.07, 1.09	27.5	
**Training status**					
Trained	5	0.37	0.09, 0.65	27.3	0.868
Recreationally active	5	0.26	−0.05, 0.56	0	
Inactive	1	0.39	−1.15, 1.93	-	
**Creatinine kinase (CK)**					
**RR dosage**					
< 600 mg/d	4	−0.91	−1.89, 0.07	90	0.838
≥600 mg/d	5	−0.79	−1.29, −0.29	31.3	
**Follow-up** ^*^					
≤ 15 min	5	−1.21	−1.80, −0.61	49.8	0.001
50 min−5h	3	−0.68	−1.40, 0.05	48.6	
≥12 h	1	0.07	−0.07, 0.21	-	
**Training duration**					
< 28 d	3	−0.43	−1.13, 0.28	75.8	0.182
≥28 d	6	−1.08	−1.72, −0.44	64.4	
**Training status**					
Trained	5	−1.36	−1.94, −0.79	54.5	0.011
Recreationally active	3	−0.29	−0.86, 0.28	63.6	
Inactive	1	−0.12	−0.92, 0.68	-	
**Malondialdehyde (MDA)**					
**RR dosage**					
< 600 mg/d	3	−0.73	−1.77, 0.30	76.7	0.086
< 600 mg/d	4	−1.73	−2.20, −1.26	0	
**Comparator type**					
Placebo	6	−0.71	−1.37, −0.05	84.5	0.333
Control	3	−1.15	−1.74, −0.56	0	
**Superoxide dismutase (SOD)**					
**RR dosage**					
< 600 mg/d	3	0.46	−0.62, 1.55	77.5	0.095
≥600 mg/d	4	1.66	0.77, 2.55	78.3	
**Total antioxidant capacity (TAC)**					
**Training duration**					
≤ 28 d	3	0.27	−0.30, 0.84	36.7	0.159
>28 d	3	0.95	0.19, 1.70	50.5	
**Lactic acid (LA)**					
**RR dosage**					
< 432 mg/d	4	−0.78	−1.90, 0.33	85.5	0.760
≥432 mg/d	3	−1.00	−1.82, −0.18	59.1	

Subgroup analyses were conducted only when at least two subgroups included three or more relatively homogeneous studies. For the variables “Duration” and “Dosage,” subgroup classification was based on the median value within each group.

^*^Time intervals were defined based on the data distribution of included studies; the gap does not affect subgroup validity.

## 4 Discussion

This meta-analysis provides strong evidence supporting the positive effects of RR supplementation on endurance performance and related biomarkers compared to placebo or control groups. Significant improvements were observed in VO_2max_, TTE, and TTP, indicating enhanced endurance capacity. RR supplementation also effectively reduced muscle damage, as evidenced by decreased CK levels, and improved antioxidant capacity, demonstrated by increased TOC and SOD levels and decreased MDA levels. However, no significant effects were observed on inflammatory biomarkers, such as IL-6 and CRP. Subgroup analysis revealed that higher doses of RR (exceeding 600 mg/day) were associated with more substantial improvements in VO_2max_. Additionally, trained individuals exhibited significantly lower CK levels compared to untrained individuals. In general, the included studies were of good quality, with 24 rated as good and 2 as moderate on the PEDro scale.

### 4.1 Effects of RR supplementation on endurance performance

As one of the key findings of this study, RR supplementation significantly enhanced VO_2max_, extended TTE, and reduced TTP compared to the control or placebo groups. These results align with prior systematic reviews (6, 7), which also highlight the beneficial effects of RR on endurance performance. By synthesizing data from multiple studies, this research provides robust statistical evidence, further supporting the potential of RR as an effective performance enhancer.

RR supplementation significantly extended TTE and reduced TTP, indicating its potential to delay fatigue onset and enhance exercise performance. These improvements are likely associated with the optimization of mitochondrial function (25, 26). Previous research suggests that RR supplementation promotes mitochondrial biogenesis and improves ATP synthesis efficiency, thereby enhancing muscle energy supply (25). Moreover, RR may further improve endurance performance by increasing fat oxidation, reducing lactate accumulation, and improving lactate clearance (9, 21). This meta-analysis provides further support by confirming that RR supplementation significantly reduces post-exercise lactate levels, further supporting the idea that RR enhances endurance performance and mitigates exercise-induced fatigue through the optimization of energy metabolism.

While RR supplementation shows promise in enhancing endurance performance, the effects observed across studies have been inconsistent, suggesting that exercise-related factors may significantly influence its effectiveness. Specifically, the study by Jówko et al. (20) did not observe significant improvements in VO_2max_ in healthy males, likely due to the absence of an exercise training component in the intervention. The lack of exercise-induced metabolic stress may have limited the potential benefits of RR supplementation. Similarly, Parisi et al. (21) found no significant effects on VO_2max_ or TTE in trained male athletes. This lack of effect may be attributed to the relatively low daily dose of RR (170 mg/day), which could have been insufficient to induce meaningful physiological changes. Furthermore, Schwarz et al. (8) found no substantial improvements in endurance performance with a lower RR dose (60 mg/day). These findings suggest that the effectiveness of RR supplementation may be sensitive to both the inclusion of exercise training and the specific dosage used. Future studies should explore the interaction between exercise training and RR supplementation, and determine the optimal dosage and duration for significant outcomes.

### 4.2 Effects of RR supplementation on antioxidant capacity, muscle damage, and inflammation

This meta-analysis indicated that RR supplementation may significantly enhance endurance performance by mitigating oxidative stress and muscle damage. Specifically, RR supplementation resulted in a marked decrease in MDA levels and a concomitant increase in TAC and SOD levels, highlighting its potential role in mitigating oxidative damage and bolstering antioxidant defense mechanisms. Oxidative stress, which frequently arises following intense exercise, leads to an accumulation of free radicals that can impair muscle cell function and delay the recovery process (27). RR supplementation appears to counteract this by enhancing the activity of key antioxidant enzymes, such as SOD and catalase, which neutralize free radicals and protect cellular integrity, thereby optimizing muscle function (28, 29). The observed decline in MDA levels, a reliable marker of lipid peroxidation, further corroborates RR's capacity to alleviate oxidative stress and protect cellular membranes. Similarly, the reduction in CK levels suggests a protective effect against exercise-induced muscle damage. While this meta-analysis did not directly evaluate energy metabolism, research suggests that antioxidants may enhance mitochondrial function and energy production, potentially facilitating more efficient recovery post-exercise (25). In summary, these findings highlight the potential of RR supplementation as a promising intervention for improving endurance performance and expediting recovery by targeting oxidative stress, supporting muscle repair, and enhancing recovery efficiency.

While RR supplementation demonstrated clear benefits in mitigating oxidative stress and muscle damage, its impact on inflammation markers such as IL-6 and CRP did not reach statistical significance. This meta-analysis indicated a trend suggesting RR's potential to reduce inflammation; however, the lack of significant findings is likely due to the limited number of studies (only three for both IL-6 and CRP) and small sample sizes. This underscores the need for further research with larger sample sizes and more rigorous methodologies to better elucidate RR's effects on exercise-induced inflammation. Therefore, although current evidence is promising, it remains inconclusive regarding RR's role in modulating post-exercise inflammatory responses.

### 4.3 Moderating effects of training factors on RR supplementation efficacy

Subgroup analysis revealed that higher doses of RR supplementation (exceeding 600 mg/day) were significantly associated with improvements in VO_2max_. This effect may be mediated through RR's ability to enhance antioxidant capacity, thereby mitigating exercise-induced oxidative stress and reducing free radical generation. Evidence suggests that reducing oxidative stress may help preserve mitochondrial structure and function, improve energy production efficiency, and delay muscle fatigue (5, 30). Furthermore, reducing oxidative stress may also improve cardiovascular function, which contributes to the enhancement of VO_2max_ (31). Therefore, dosages exceeding 600 mg/day may offer potential benefits for optimizing endurance performance. However, it is important to note that the optimal dosage could vary based on individual characteristics and specific training contexts. Future research should aim at larger, well-controlled trials to establish the most effective and safe dosage range for diverse populations.

Subgroup analysis also revealed a significant difference in CK levels between trained and untrained individuals following RR supplementation, with trained individuals exhibiting lower CK levels. This suggests that individuals with higher baseline fitness levels may derive greater benefits from RR supplementation in reducing exercise-induced muscle damage. Trained individuals typically possess more efficient recovery mechanisms, which may enhance their response to RR, thereby optimizing its effectiveness in mitigating muscle damage and promoting faster recovery (32, 33). Thus, when assessing the potential benefits of RR supplementation, it is essential to consider an individual's training status. Tailoring supplementation strategies to an individual's fitness level could optimize outcomes for both athletes and recreational exercisers.

Moreover, the follow-up time point significantly moderated the effect of RR on CK levels. Measurements taken within 15 min post-exercise showed a more pronounced reduction in CK compared to later time points. This may be explained by the rapid antioxidant and anti-inflammatory effects of RR's active compounds early after exercise, which effectively reduce acute muscle damage and inhibit CK release (5). As time progresses, CK is gradually released and influenced by various physiological processes, diminishing the supplement's protective effects (34). In contrast, intervention duration did not show a significant moderating effect on endurance performance or related biomarkers. This lack of effect may be due to the relatively short durations of most included studies, with the majority lasting < 6 weeks and only three studies exceeding this timeframe. Such limited variation reduces the ability to detect duration-dependent effects. Future research should prioritize monitoring physiological markers within 15 min post-exercise and consider extending intervention duration to better understand RR's effects on muscle recovery and endurance performance.

None of the included studies reported adverse effects related to supplementation. While this suggests a favorable safety profile, underreporting cannot be ruled out. Future studies should explicitly report adverse events to enable a comprehensive evaluation of both efficacy and safety.

### 4.4 Limitation

This meta-analysis review has certain limitations. First, only one analysis included more than 10 studies, while the rest had fewer, which may limit the precision of effect estimates and increase uncertainty. Second, data constraints prevented the inclusion of key biomarkers such as glutathione peroxidase, thiobarbituric acid reactive substances, and myoglobin, reducing the scope of findings. Lastly, while subgroup analyses were performed to address high heterogeneity, yet residual variability persisted owing to differences in study designs, limited numbers of studies with small sample sizes, and incomplete reporting of training protocols. These limitations may affect the robustness and generalizability of the findings. Future studies should prioritize larger sample sizes, standardized protocol reporting, and comprehensive biomarker assessment.

## 5 Conclusions

This meta-analysis provides strong evidence supporting the positive effects of RR supplementation on endurance performance, oxidative stress, muscle damage, and metabolic efficiency. RR supplementation significantly improved endurance outcomes, including increased VO_2max_, prolonged TTE, and reduced TTP. It also enhanced antioxidant capacity, evidenced by increased TAC and SOD levels, and decreased MDA levels. Muscle damage markers, such as CK, were significantly reduced, and lactate levels dropped, suggesting improved metabolic efficiency. However, RR supplementation did not show significant effects on exercise-induced inflammation, as measured by IL-6 and CRP, likely due to the limited number of studies evaluating these outcomes.

## Data Availability

The original contributions presented in the study are included in the article/Supplementary material, further inquiries can be directed to the corresponding author.

## Supplementary material

Supplementary File 1 Search alert.

Supplementary File 2 Physiotherapy Evidence Database (PEDro) scale ratings.

Supplementary File 3 Funnel plots.

Supplementary File 4 Forest plots.
