# Supplementary material for: The effect of Rhodiola rosea supplementation on endurance performance and related biomarkers: a systematic review and meta-analysis
Source: Front Nutr. 2025 Sep 25;12:1645346. doi: 10.3389/fnut.2025.1645346 (PMC12507841; doi:10.3389/fnut.2025.1645346)
Supplement: Supplementary File 1 — Search alert. [file Table_1.DOCX]

**Supplementary File 1.** Search Alert

Web of Science (122)

[Topic] ("Rhodiola rosea" OR "Rhodiola" OR "Rosea" OR "Roseroot" OR "Golden root" OR "Arctic root" OR "Rhodioloside" OR " Salidroside") AND [Topic] ("Endurance" OR“Sport” OR “Athletic” OR “exercise” OR “training”)

Pubmed (87)

("Rhodiola rosea"[Title/Abstract] OR "Rhodiola"[Title/Abstract] OR "Rosea"[Title/Abstract] OR "Roseroot"[Title/Abstract] OR "Golden root"[Title/Abstract] OR "Arctic root"[Title/Abstract] OR "Rhodioloside"[Title/Abstract] OR " Salidroside"[Title/Abstract]) AND ("Endurance" OR"Sport"[Title/Abstract] OR "Athletic"[Title/Abstract] OR "exercise"[Title/Abstract] OR "training"[Title/Abstract])

Scopus (199)

AB ("Rhodiola rosea" OR "Rhodiola" OR "Rosea" OR "Roseroot" OR "Golden root" OR "Arctic root" OR "Rhodioloside" OR " Salidroside") AND AB ("Endurance" OR“Sport” OR “Athletic” OR “exercise” OR “training”)

EBSCO MEDICAL Databases (161)

Search Alert: AB ("Rhodiola rosea" OR "Rhodiola" OR "Rosea" OR "Roseroot" OR "Golden root" OR "Arctic root" OR "Rhodioloside" OR " Salidroside") AND AB ("Endurance" OR“Sport” OR “Athletic” OR “exercise” OR “training”)

CNKI (239)

[Topic] (“Hong jing tian”) AND [Topic] (“yun dong”)

**Supplementary File 2.**

**Table S1.** Physiotherapy Evidence Database (PEDro) scale ratings

| **References** | **Items*** | | | | | | | | | | | **Total**  **(out of 10)** |
| --- | --- | --- | --- | --- | --- | --- | --- | --- | --- | --- | --- | --- |
|  | **1** | **2** | **3** | **4** | **5** | **6** | **7** | **8** | **9** | **10** | **11** |  |
| Abidov et al., 2004 | 1 | 1 | 0 | 1 | 1 | 0 | 1 | 1 | 1 | 1 | 1 | 8 |
| Chen et al., 2014 | 1 | 1 | 0 | 0 | 1 | 0 | 1 | 1 | 1 | 1 | 1 | 7 |
| Cui et al., 2001 | 1 | 1 | 0 | 0 | 0 | 0 | 0 | 1 | 1 | 1 | 1 | 5 |
| de Bock et al., 2004 | 1 | 0 | 0 | 1 | 1 | 0 | 0 | 1 | 1 | 1 | 1 | 7 |
| Duncan et al., 2015 | 1 | 0 | 0 | 1 | 1 | 0 | 1 | 1 | 1 | 1 | 1 | 7 |
| Gao & Zhang 1996 | 1 | 1 | 0 | 1 | 1 | 0 | 1 | 1 | 1 | 1 | 1 | 8 |
| He 2007 | 1 | 1 | 0 | 1 | 0 | 0 | 0 | 1 | 1 | 1 | 1 | 6 |
| Jia et al., 2021 | 1 | 0 | 0 | 1 | 0 | 0 | 0 | 1 | 1 | 1 | 1 | 5 |
| Jowko et al., 2018 | 1 | 1 | 0 | 0 | 1 | 1 | 1 | 1 | 1 | 1 | 1 | 8 |
| Kreipke et al., 2021 | 1 | 1 | 0 | 1 | 1 | 0 | 1 | 1 | 1 | 1 | 1 | 8 |
| Liao et al., 2019 | 1 | 1 | 0 | 1 | 1 | 1 | 1 | 1 | 1 | 1 | 1 | 9 |
| Lin et al., 2019 | 1 | 0 | 0 | 0 | 1 | 0 | 1 | 1 | 1 | 1 | 1 | 6 |
| Noreen et al., 2009 | 1 | 0 | 0 | 0 | 1 | 0 | 1 | 1 | 1 | 1 | 1 | 6 |
| Noreen et al., 2013 | 1 | 0 | 0 | 0 | 1 | 0 | 1 | 1 | 1 | 1 | 1 | 6 |
| Parisi et al., 2009 | 1 | 0 | 0 | 0 | 1 | 0 | 1 | 1 | 1 | 1 | 1 | 6 |
| Parisi et al., 2010 | 1 | 0 | 0 | 0 | 1 | 0 | 1 | 1 | 1 | 1 | 1 | 6 |
| Qiao et al., 2009 | 1 | 1 | 0 | 1 | 1 | 0 | 0 | 1 | 1 | 1 | 1 | 7 |
| Schwarz et al., 2024 | 1 | 1 | 1 | 0 | 1 | 0 | 1 | 1 | 1 | 1 | 1 | 8 |
| Shanely et al., 2014 | 1 | 1 | 0 | 1 | 1 | 0 | 1 | 1 | 1 | 1 | 1 | 8 |
| Skarpanska et al., 2009 | 1 | 1 | 0 | 1 | 1 | 0 | 1 | 1 | 1 | 1 | 1 | 8 |
| Song et al., 2015 | 1 | 1 | 0 | 1 | 1 | 0 | 0 | 1 | 1 | 1 | 1 | 7 |
| Timpmann et al., 2018 | 1 | 1 | 0 | 0 | 1 | 0 | 1 | 1 | 1 | 1 | 1 | 7 |
| Yun et al., 2024 | 1 | 1 | 0 | 0 | 1 | 0 | 1 | 1 | 1 | 1 | 1 | 7 |
| Zhang et al., 2009 | 1 | 1 | 0 | 1 | 1 | 0 | 1 | 1 | 1 | 1 | 1 | 8 |
| Zhao 2018 | 1 | 1 | 0 | 1 | 1 | 0 | 1 | 1 | 1 | 1 | 1 | 8 |
| Zheng et al., 2023 | 1 | 1 | 0 | 1 | 0 | 0 | 0 | 1 | 1 | 1 | 1 | 8 |
| Median score = 7 | | | | | | | | | | | | |

Note: * Detailed explanations for PEDro scale items are available at https://pedro.org.au/English/resources/pedro-scale/ (access for this review: February 12, 2025).

**Supplementary File 3.** Funnel Plots


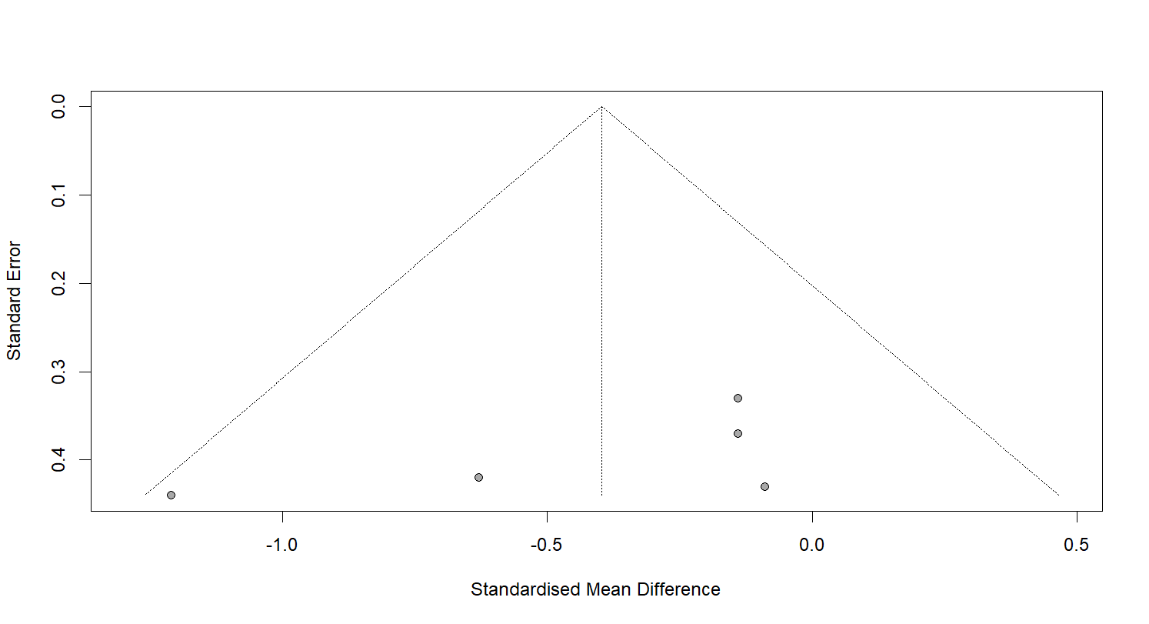


Figure 1. Time trial performance (TTP) funnel plot

**
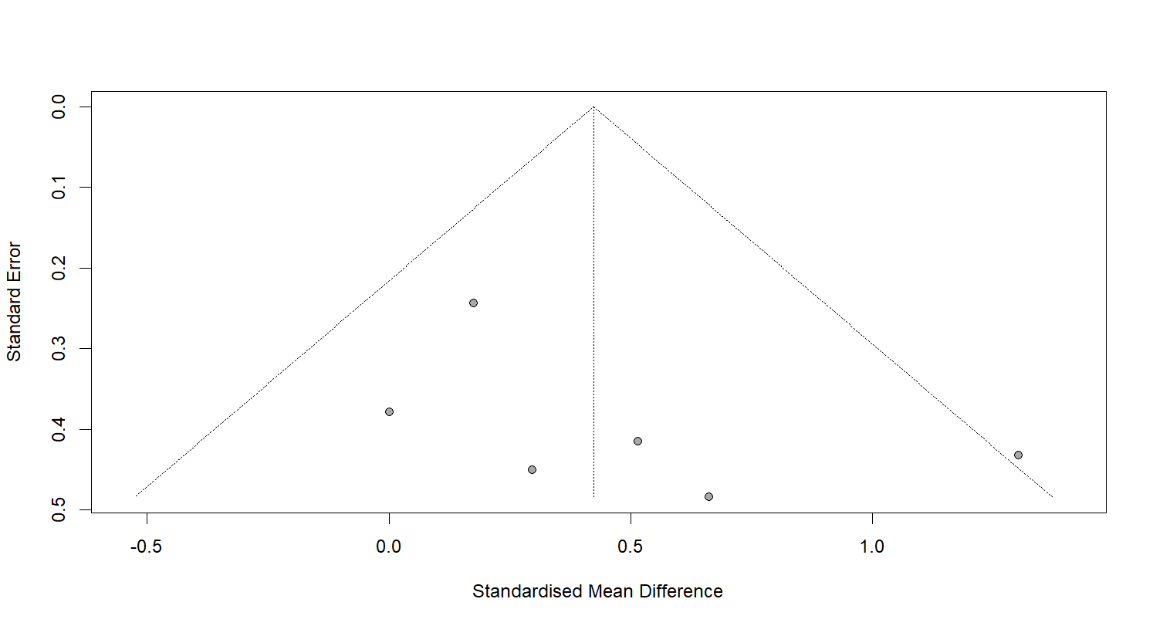
**

Figure 2. Time to exhaustion (TTE) funnel plot

**
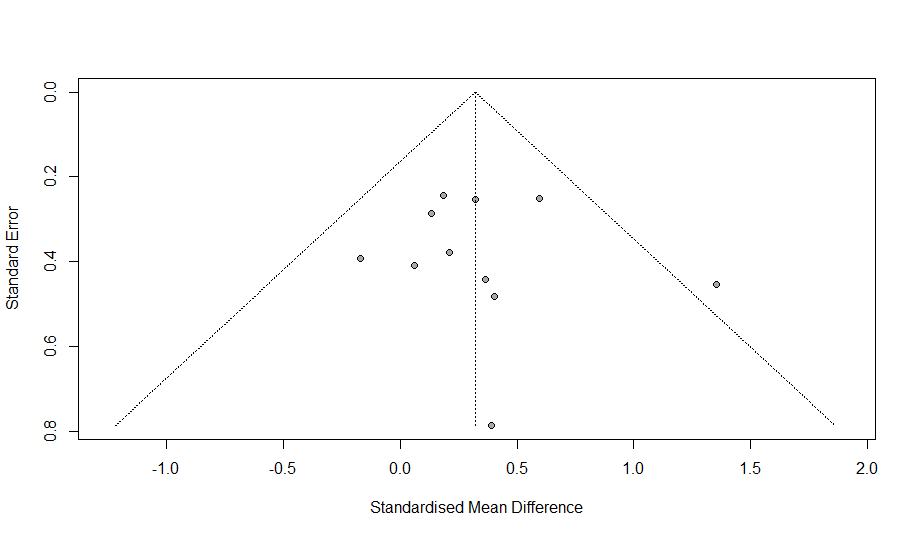
**

Figure 3. VO2max funnel plot

**
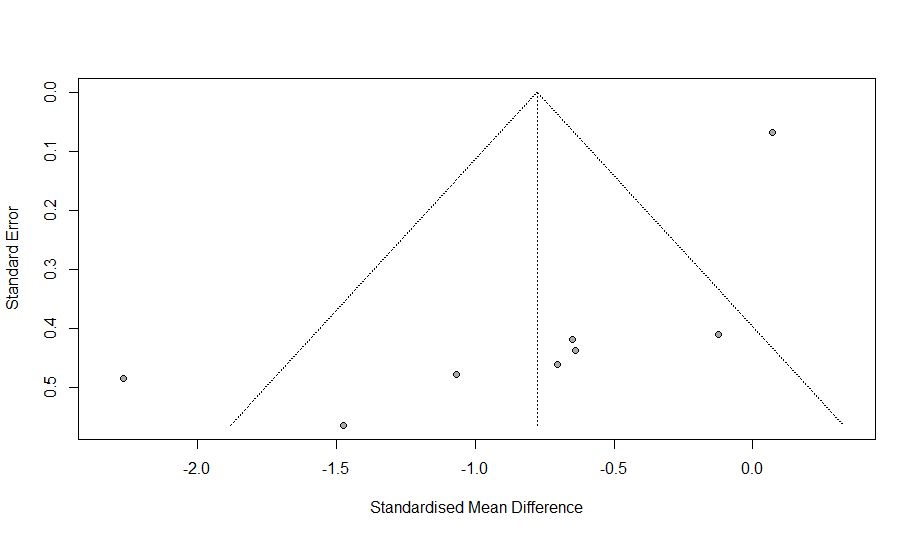
**

Figure 4. Creatinine kinase (CK) funnel plot

**
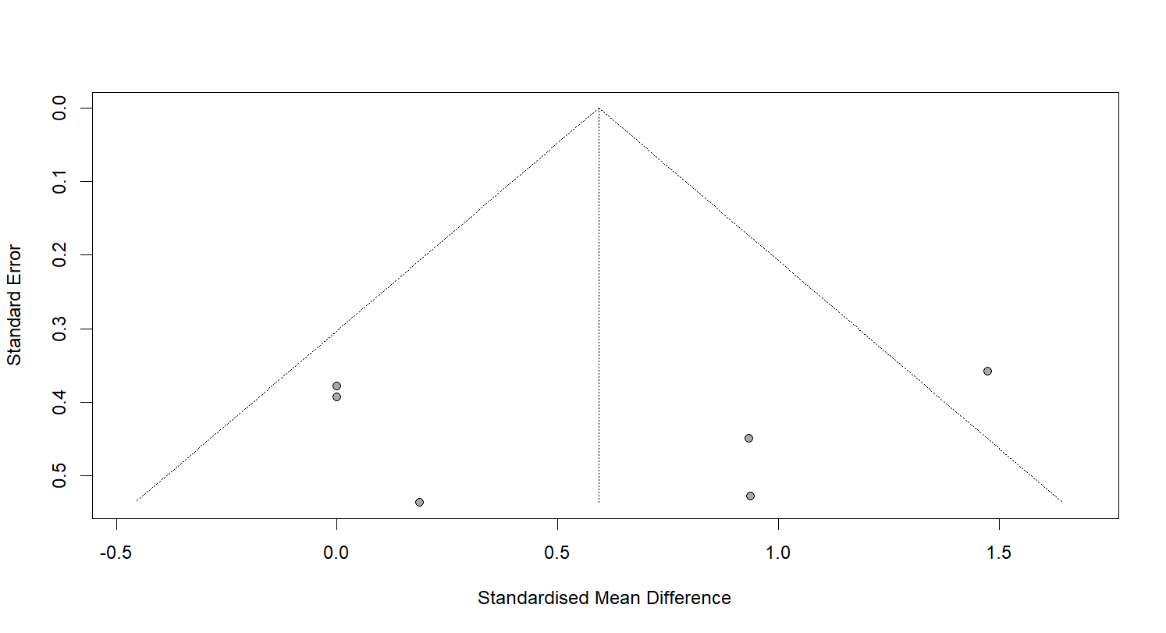
**

Figure 5. Total antioxidant capacity (TAC) funnel plot

**
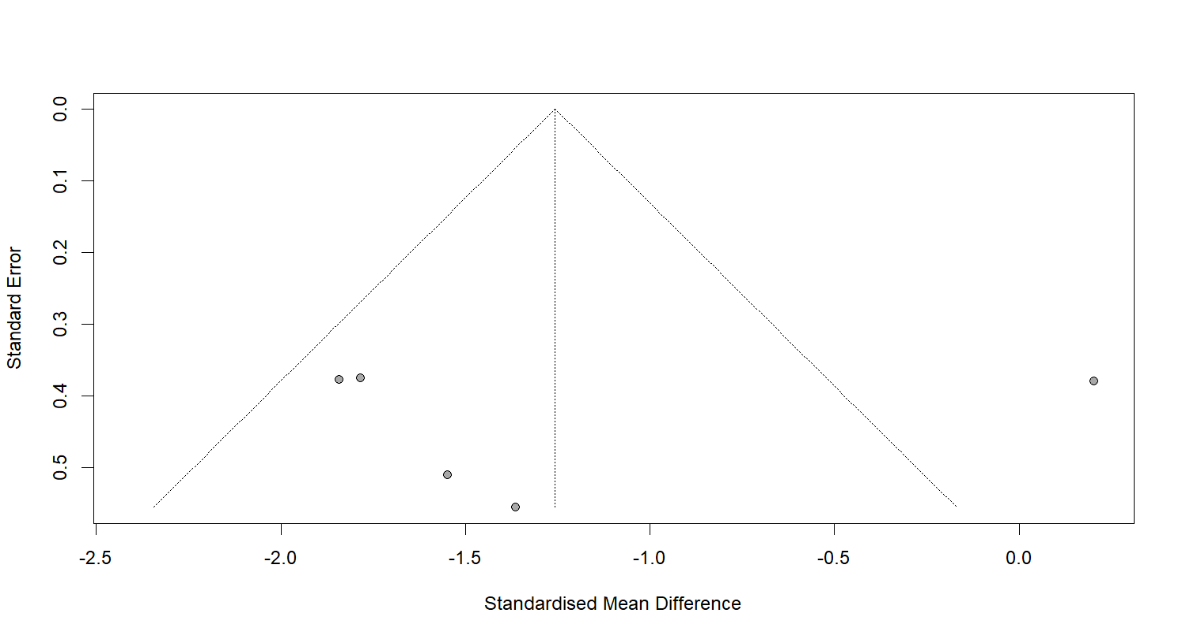
**

Figure 6. Malondialdehyde (MDA) funnel plot

**
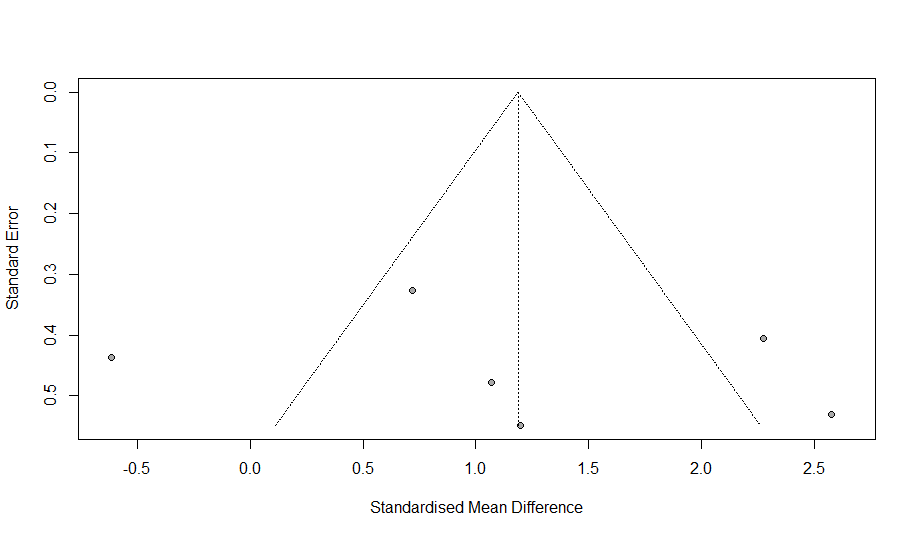
**

Figure 7. Superoxide dismutase (SOD) funnel plot

**
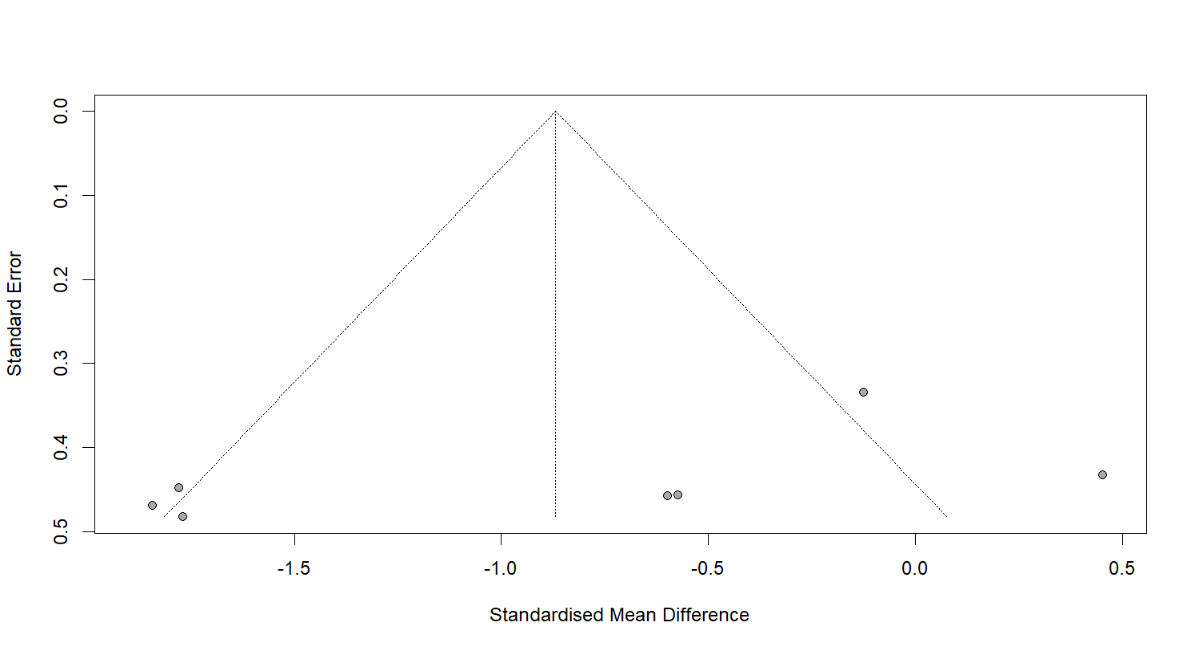
**

Figure 8. Lactic acid (LA) funnel plot

**Supplementary File 4.** Forest Plots

**
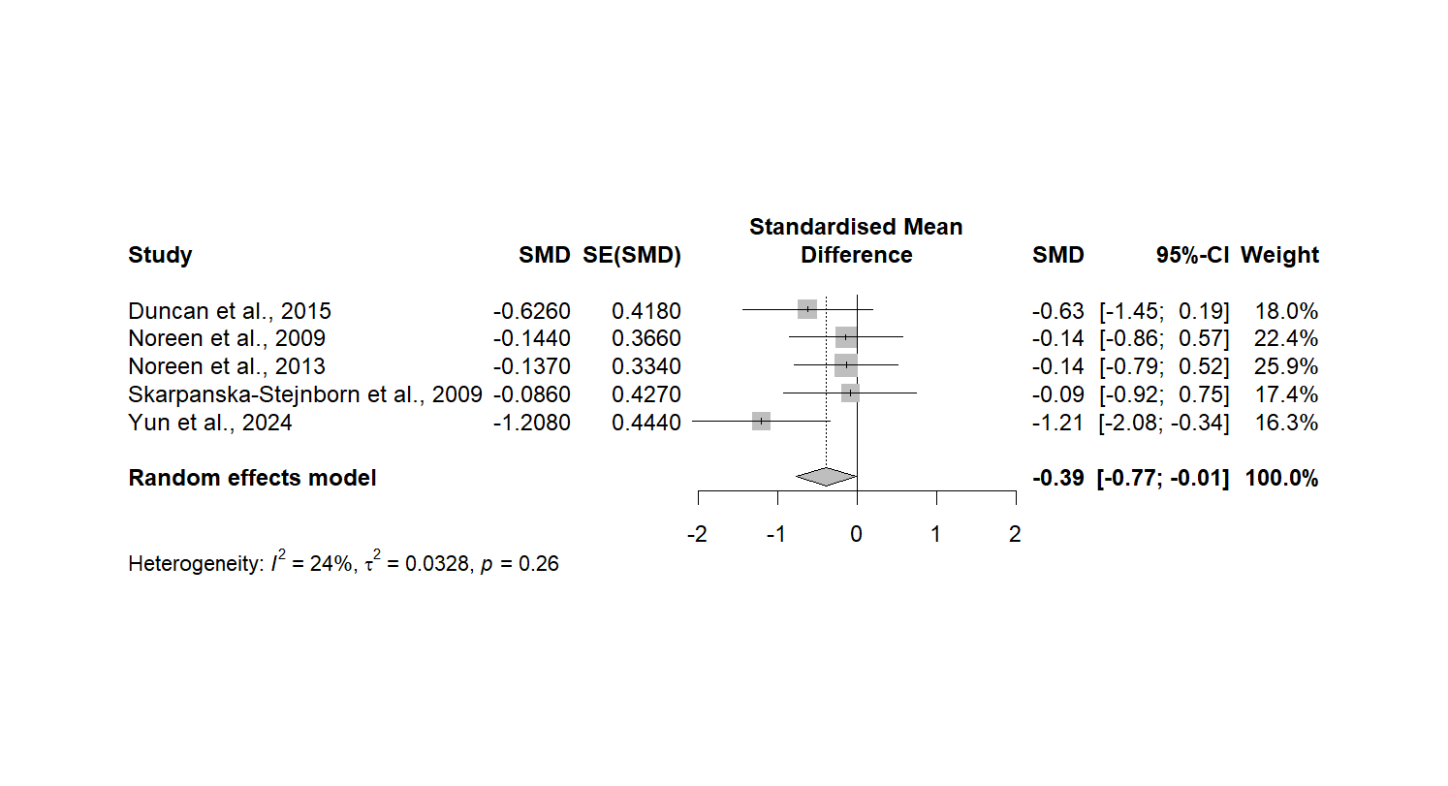
**

Figure 1. Time trial performance (TTP) forest plot


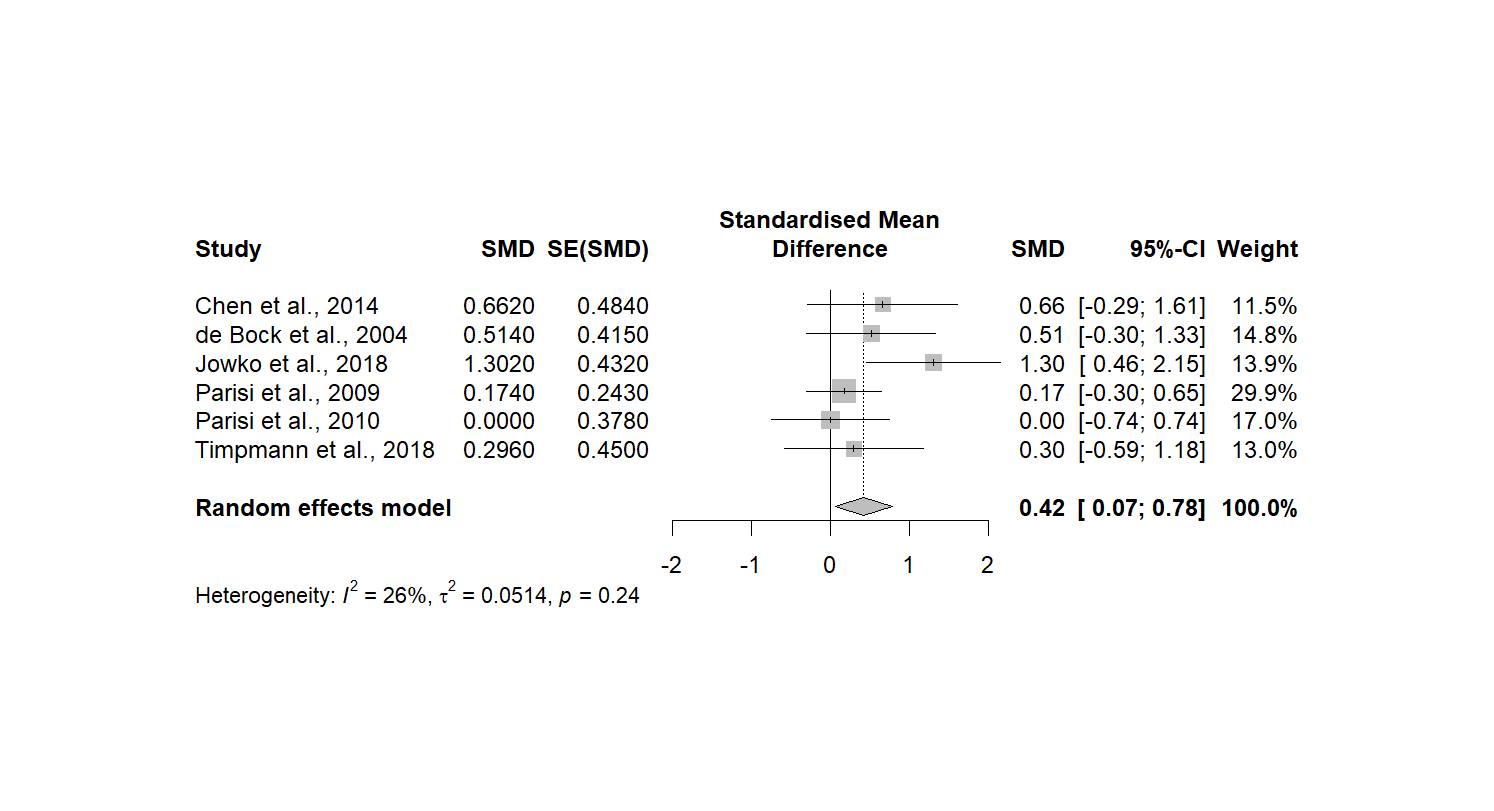


Figure 2. Time to exhaustion (TTE) forest plot


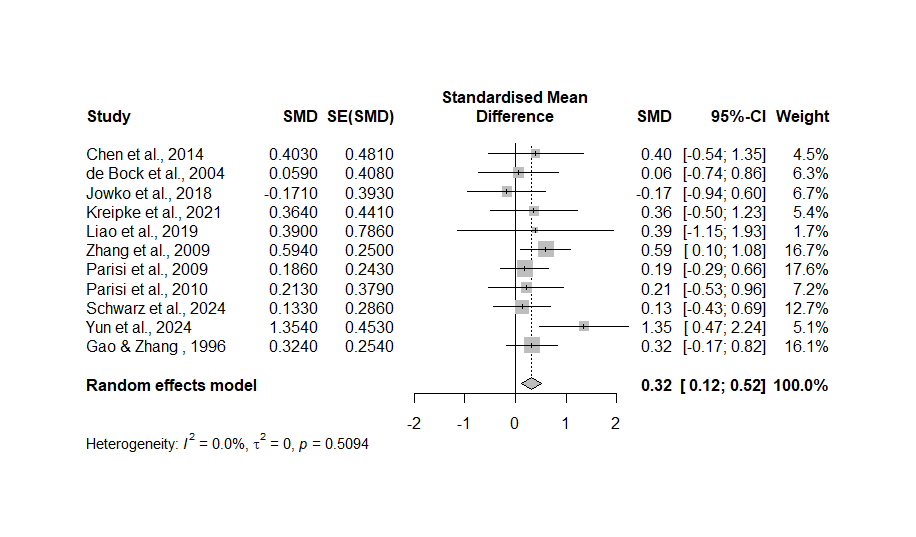


Figure 3. VO_2max_ forest plot


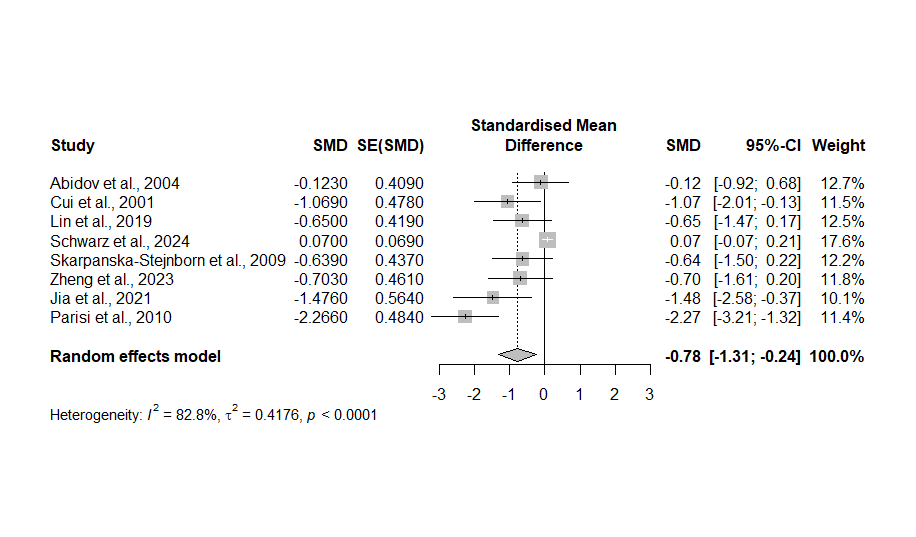


Figure 4. Creatinine kinase (CK) forest plot


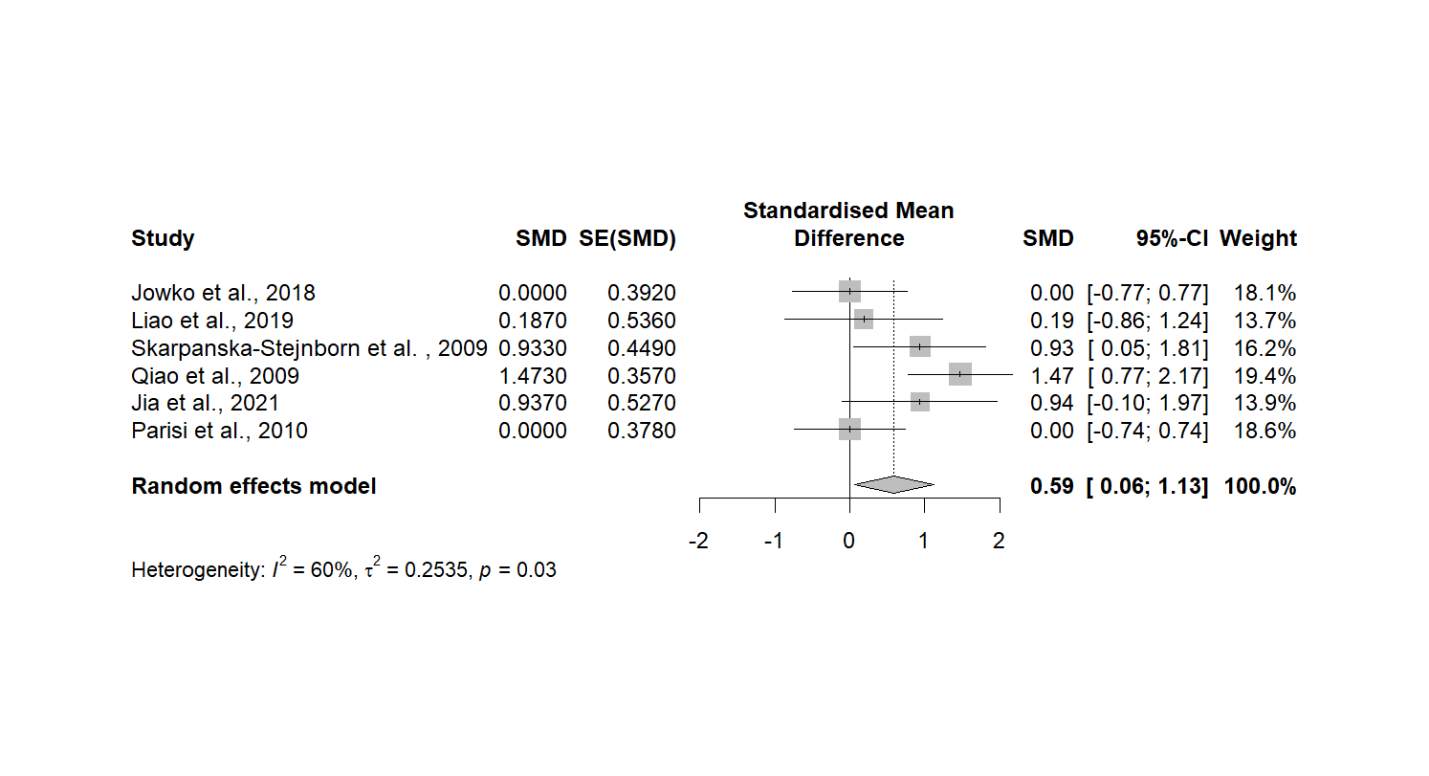


Figure 5. Total antioxidant capacity (TAC) forest plot


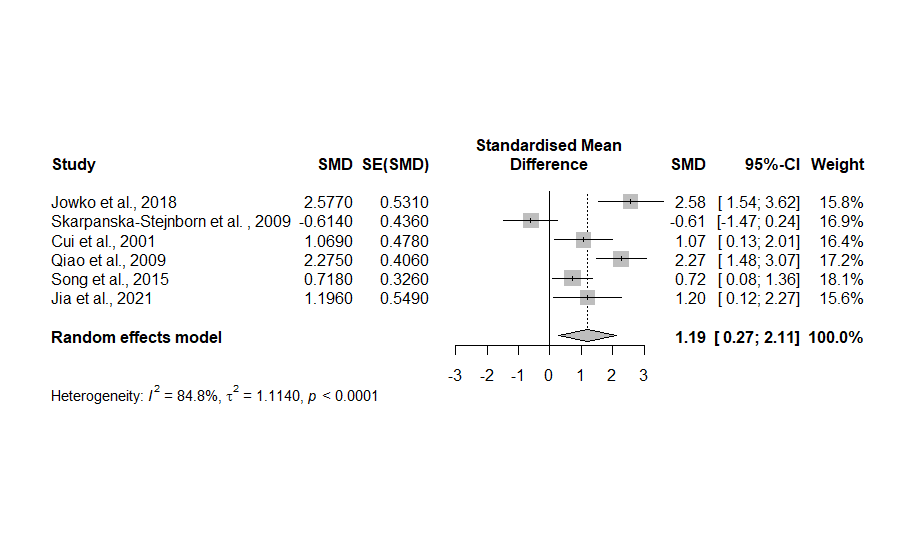


Figure 6. Superoxide dismutase (SOD) forest plot


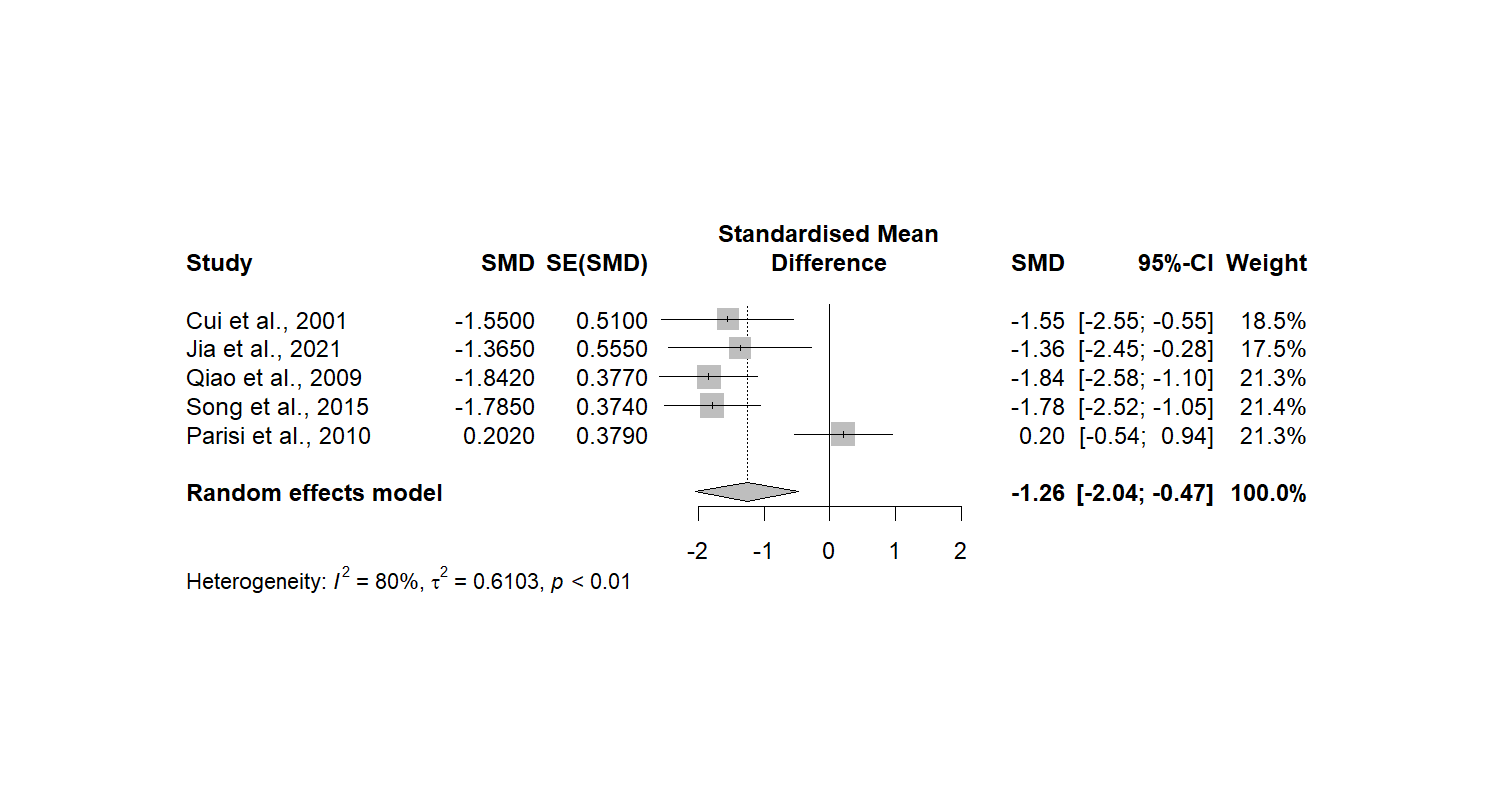


Figure 7. Malondialdehyde (MDA) forest plot


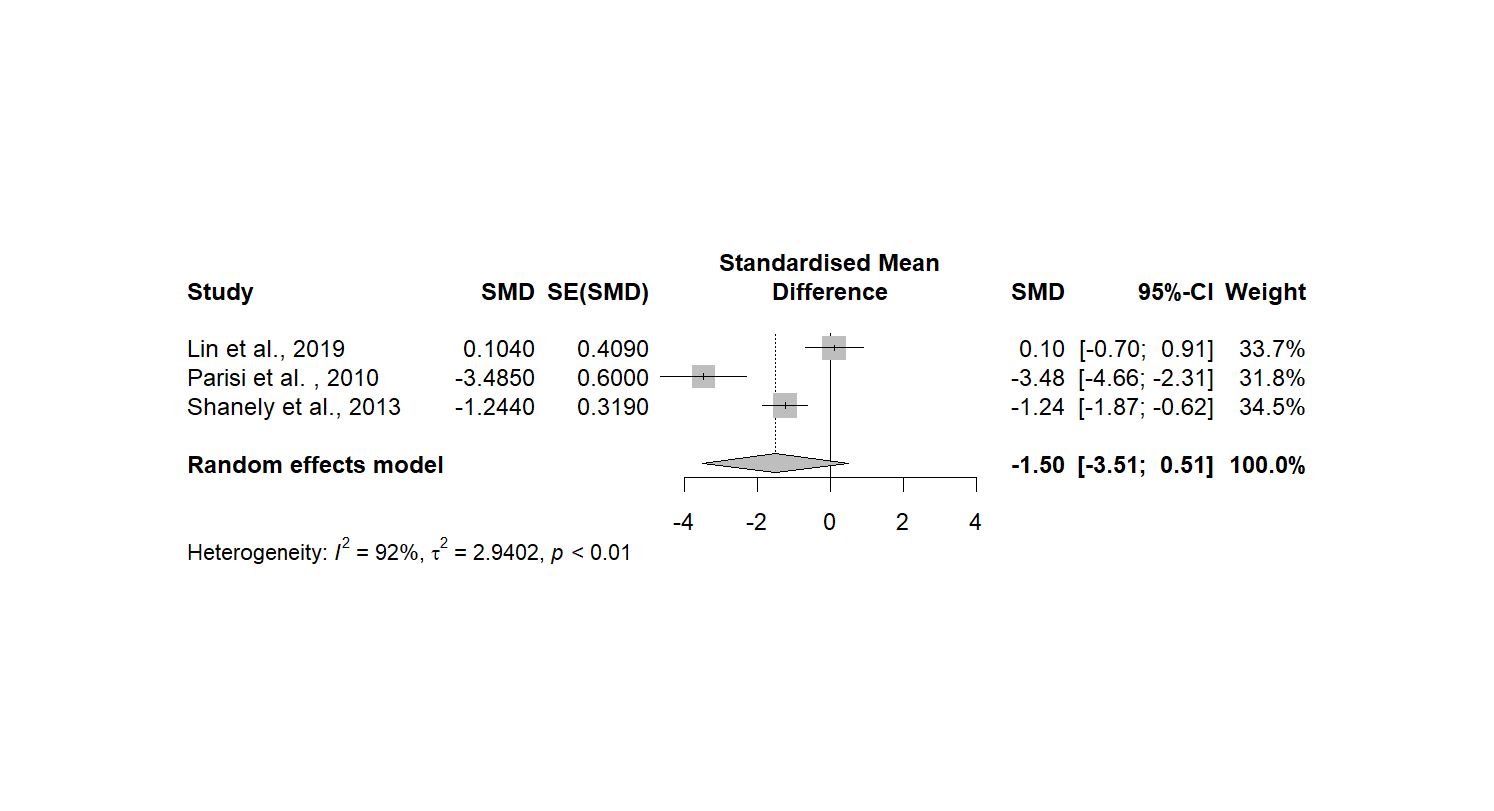


Figure 8. Interleukin-6 (IL-6) forest plot


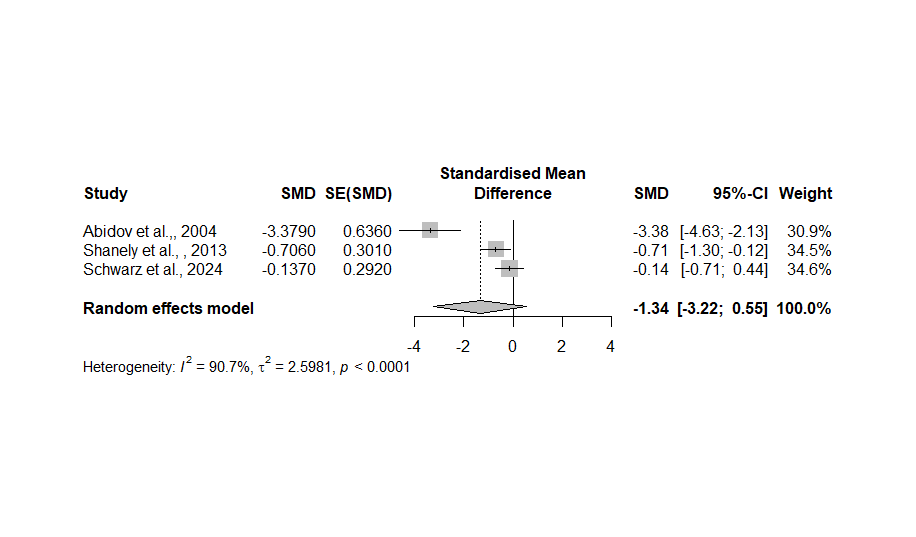


Figure 9. C-reactive protein (CRP) forest plot


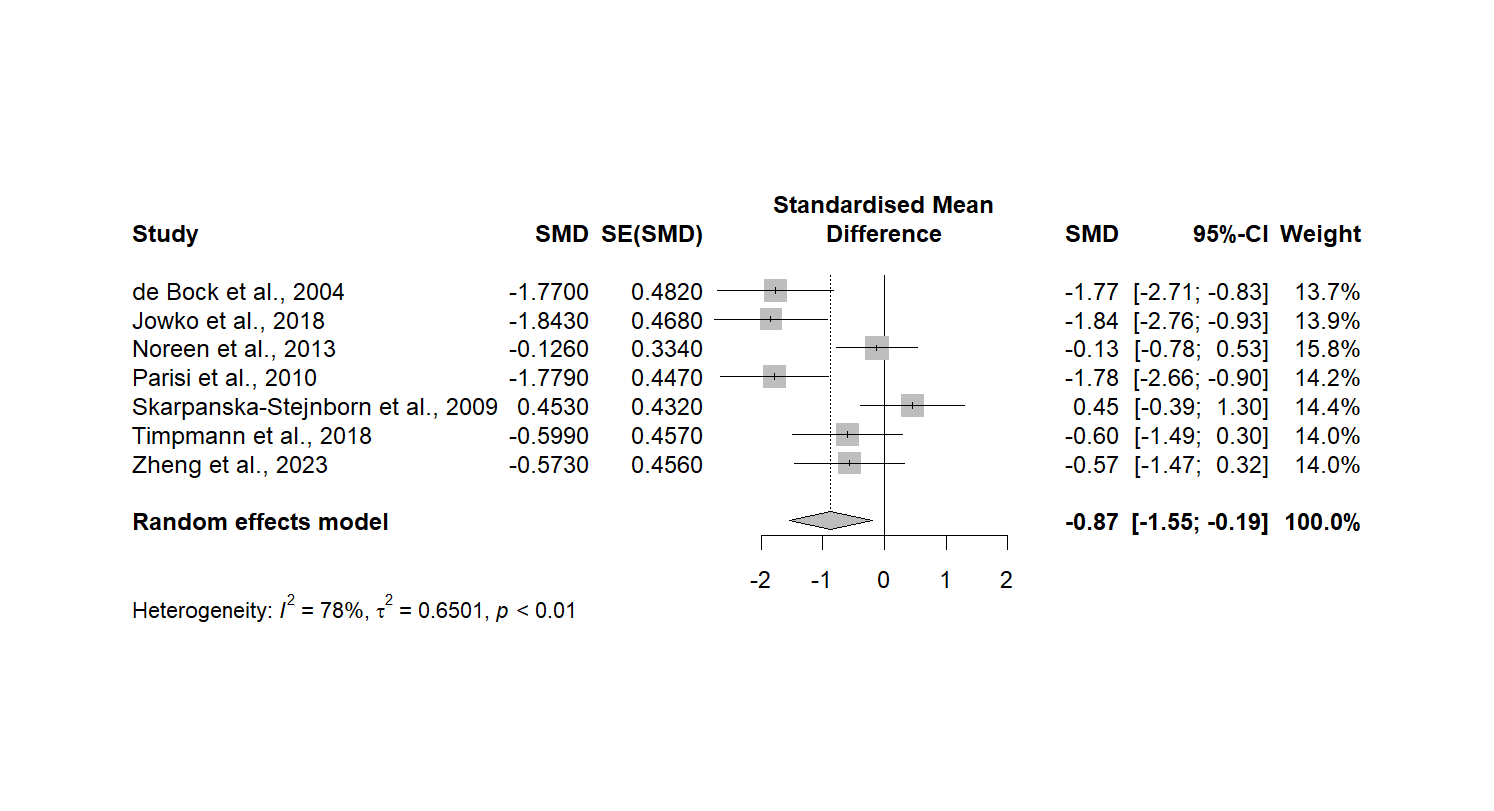


Figure 10. Lactic acid (LA) forest plot
